# Comparison of joint status using ultrasound assessments and Haemophilia Joint Health Score 2.1 in children with haemophilia

**DOI:** 10.3389/fmed.2023.1193830

**Published:** 2023-07-18

**Authors:** Yanju Li, Feiqing Wang, Chengyun Pan, Jing Zhang, Qian Zhang, Lingying Ban, Lingling Song, Jishi Wang, Zhixu He, Xiaojing Zeng, Dongxin Tang, Yang Liu

**Affiliations:** ^1^Department of Hematology Oncology, Affiliated Hospital of Guizhou Medical University, Guiyang, China; ^2^Clinical Medical Research Center, The First Affiliated Hospital of Guizhou University of Traditional Chinese Medicine, Guiyang, China; ^3^Academy of Medical Engineering and Translational Medicine, Tianjin University, Tianjin City, China; ^4^Key Laboratory of Adult Stem Cell Translational Research, Chinese Academy of Medical Sciences, Guizhou Medical University, Guiyang, China

**Keywords:** Haemophilia Joint Health Score (HJHS), haemophilia, arthropathy, ultrasonography, children

## Abstract

**Introduction:**

Ultrasound (US) has gained popularity in the evaluation of haemophilic joint diseases because it enables the imaging of soft-tissue lesions in the joints and bone-cartilage lesions. We aimed to determine the correlation between US evaluations and clinical assessments performed using HJHS 2.1 and to evaluate their respective characteristics in assessing early haemophilic arthropathy.

**Methods:**

A total of 178 joints (32 knees, 85 elbows, and 61 ankles) in 45 haemophilia A patients (median age, 10 years; range, 6–15) were assessed using US and HJHS 2.1. Ultrasonographic scoring was performed in consensus assessments by one imager by using the US scores.

**Results:**

The total HJHS 2.1 and US scores showed a strong correlation (rS=0.651, *P*=0.000, CI: 0.553–0.763), with an excellent correlation for the elbows (rS=0.867, *P*=0.000, CI: 0.709–0.941) and a substantial correlation for the knees (rS=0.681, *P*=0.000, CI: 0.527–0.797). The correlation for the ankles was relatively moderate (rS=0.518, P=0.000, CI: 0.308–0.705). Nine subjects (15.5%) without abnormalities, as indicated by HJHS 2.1, showed haemophilic arthropathy in US scoring. All nine joints showed moderate (1/9) to severe (8/9) synovial thickening in the ankle (5/9) and elbow joints (4/9). In contrast, 50 joints (50.5%) showed normal US scores and abnormal changes as indicated by HJHS 2.1. S scores correlated well with HJHS 2.1 for overall and individual joints.

**Discussion:**

US could identify some early pathological changes in joints showing normal clinical findings, but still cannot replace the HJHS; however, it can serve as an imaging examination complementing HJHS 2.

## 1. Introduction

Haemophilia is a common sex-chromosome recessive hereditary haemorrhagic disease caused by a deficiency of coagulation factor VIII or IX. Joint bleeding is the most commonly reported type of haemorrhage in haemophilia patients, which could lead to synovial hypertrophy and direct bleeding-related osteochondral changes. The elbows, knees, and ankles are the most affected among all joints in haemophilia. With the widespread use of prophylaxis, the onset of arthropathy has significantly reduced (1). However, because of many reasons, the prevention and treatment of haemophilia in China, especially in Guizhou Province, are lagging behind those in developed countries and the incidence of joint dysfunction is high, which seriously affects the quality of life of affected patients. Hence, early periodic monitoring of joint lesions in haemophilia patients is recommended, which is aimed at identifying early arthropathic changes and prevention of the development or progression of haemophilic arthropathy.

The Haemophilia Joint Health Score (HJHS 2.1) was developed to detect early joint changes in moderate or severe haemophilia in patients aged higher than 3 years; however, it did not consider normal childhood variations. Considering that physical examination assessment scores lack the sensitivity and specificity required for the identification of early and subclinical joint abnormalities, radiography has been recommended in addition to a clinical examination for assessing the joint status and disease progression in haemophilia patients.

Magnetic resonance imaging (MRI) is an accurate routine imaging method for joint lesions in haemophilia patients (2); however, its application is limited due to its high cost, the time required, and the possible requirement of sedative drugs. It can be used as a routine imaging examination method for screening and follow-up in only a few haemophilia patients with special needs. Ultrasound (US) examination, an imaging method that allows the imaging of soft-tissue lesions in the joints and bone-cartilage lesions, is becoming popular in the evaluation and examination of haemophilic joint diseases in recent years because it is convenient, economical, and safe (3, 4). Several previous studies have investigated the correlation between US findings and HJHS (5–7); however, the sample sizes in these studies were relatively small, and as of now, consensus on the issue is lacking. Hence, this study was aimed at assessing the value of US in the assessment of joint status in haemophilia by comparing it with HJHS 2.1 in children with haemophilia.

## 2. Materials and methods

### 2.1. Research object

A total of 178 knee joints of 45 children with haemophilia in the Affiliated Hospital of Guizhou Medical University in Guizhou Province were selected; all of the patients were male and aged between 6 and 15 years. All cases met the 2014 diagnostic criteria of the consensus of Chinese experts for the diagnosis and treatment of haemophilia A (8). Consent was obtained from all patients and/or their families prior to participation in this study.

### 2.2. Inspection method

*Equipment:* US: Low-speed blood flow conditions of the skeletal muscle were selected using the Philips iU22 colour US instrument with a high-frequency line array probe (5 ~ 12 MHz). Different positions were imaged according to the different joints of the patients. Images were obtained in the sitting position or by lying on the front and sides of the joint. The posterior parts of the joints were examined in the prone position. Random images of the joints were obtained in the sagittal and coronal views.

*HJHS:* Clinical function was assessed by a physiotherapist according to HJHS version 2.1. The HJHS 2.1 evaluates potential joint swelling (scale 0–3), swelling duration (scale 0–1), muscle atrophy (scale 0–2), crepitus during activity (scale 0–2), decreased curvature (scale 0–3), decreased stretch (scale 0–3), joint pain (scale 0–2), and loss of strength (scale 0–4). The HJHS for joint level ranges from 0 points, indicating perfect clinical function, to 20 points, indicating a severe loss of clinical function.

*Ultrasound examination:* US examination was performed on the same day by two imaging specialists. They observed for the following parameters: exudation (joint effusion or haemorrhage) (scale 0–3), fibrous septum (scale 0–1), synovial thickness (normal value: <1 mm) (scale 1–3), synovial thickening with synovial vascular hyperplasia (scale 1–2), hemosiderin deposition (scale 0–3), cartilage changes (scale 1–3), bone erosion (irregular bone mass destruction) (scale 0–1), osteophytes (formation of bone hyperplasia at the edge of the joint) (scale 0–1), and bone reconstruction (irregular and inconsistent joint surfaces) (scale 0–1). The total score possible is 18.

### 2.3. Statistical analysis

Data were analysed using SPSS l8.0 statistical software. Spearman’s rank of correlation coefficient (rS) was used to study the correlations of the outcome measurement scores. A correlation was considered poor if rS was <0.4, moderate if rS was 0.4–0.6, good or substantial if rS was 0.6–0.8, and excellent if rS was >0.8.

## 3. Results

In our study, US-based scoring was performed for two of the three joints of the elbow, knee, and ankle of each patient. In total, 178 joints of 45 patients were assessed. Two joints (one knee and one ankle) could not be assessed because the data collected were incomplete. The median age of these patients was 10 years (range 6–15). The median HJHS and US score were 2 (range 0–17) and 0 (range 0–13) respectively. The baseline characteristics of the study joints are shown in Table 1.

**Table 1:** Patients’ baseline characteristics (*n* = 45 patients, 178 joints).

	Median (25th–75th percentile) or *n* (%)	Range
Age (years)	10 (6–15)	3–17
Elbow (*n* = 32 joints)		
HJHS	2.5 (0–10)	0–16
US	4 (0–7)	0–12
Knee (*n* = 85 joints)		
HJHS	2 (0–9.5)	0–17
US	0 (0–5)	0–13
Ankle (*n* = 61 joints)		
HJHS	2 (0–5)	0–13
US	0 (0–4)	0–11
Total (*n* = 178 joints)		
HJHS	2 (0–7)	0–17
US	0 (0–5)	0–13

HJHS, Haemophilia Joint Health Score; US, ultrasound.

**Figure 1:**
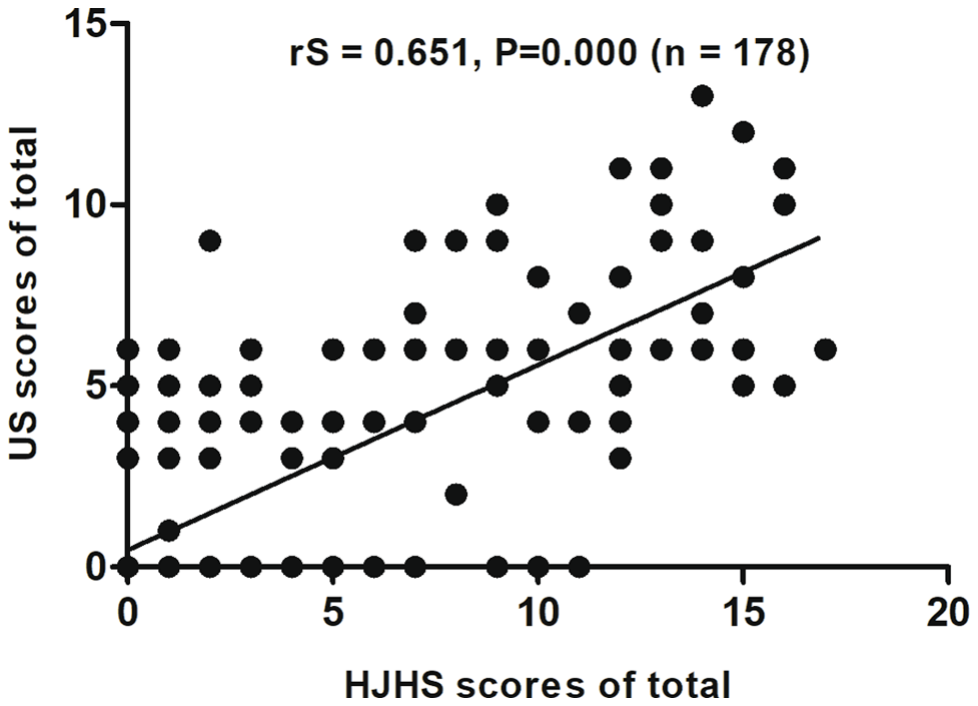
Scatter plot showing the correlation between the total clinical Hemophilia Joint Health Score (HJHS 2.1) and ultrasound (US) scores.

Among these 178 joints, the HJHS 2.1 was positive for 120 joints (67.4%) and US scores for 79 joints (44.4%). There was a good correlation between HJHS 2.1 and US scores for all study joints (rS = 0.651, *p* = 0.000, CI: 0.553–0.763) (Figure 1). Nine subjects (15.5%, 9/58 joints) without abnormalities, as indicated by the HJHS 2.1, were found to have haemophilic arthropathy based on US scores (3 points for three patients, 4 points for four patients, 5 points for one patient and 6 points for one patient, respectively). Moderate (1/9) to severe (8/9) synovial thickening in the ankle joints (5/9) and elbow joints (4/9) was noted in all of these nine cases by using US. In seven of these cases, synovial thickening with synovial vascular proliferation in the ankle joints (3/7) and elbow joints (4/7) were noted. One case showed small changes in the cartilage of the elbow joint and the other case showed a small amount of hemosiderin deposition in the ankle joint, based on synovial thickening and synovial thickening with synovial vascular proliferation that were not detected by clinical examination. US scores according to HJHS 2.1 are shown in Table 2. Fifty joints (50.5%, 50/99 joints) had normal US scores but abnormal changes based on the HJHS 2.1 were found, which included 1 point for nine joints, 2 points for 17 joints, 3 points for seven joints, 4 points for seven joints, 5 points for two joints, 6 points for three joints, 7 points for two joints, 9 points for one joint 10 points for one joint, and 11 points for one joint. All of these patients had at least one with muscle atrophy, joint strength changes or swelling, partial joints with swelling for ≥6 months combined with or without joint pain, crepitus during activity, and decreased curvature or decreased stretch that could not be identified using the US scores.

**Table 2:** Ultrasound findings according to HJHS 2.1 results at the joint level.

HJHS 2.1 score	Number of patients	Median US score (25th–75th percentile)	US score = 0 *n* (%)	US score = 18 *n* (%)
0	58	0 (0–0)	49 (84.5)	0 (0)
1-3	48	0 (0–3)	33 (68.8)	0 (0)
4-6	24	1.5 (0–4)	12 (50)	0 (0)
7-9	16	5.5 (2.5–8.5)	3 (18.8)	0 (0)
10-12	15	6.0 (4–8)	2 (13.3)	0 (0)
>12	17	9.0 (6–11)	2 (11.8)	0 (0)

HJHS, Haemophilia Joint Health Score, range 0–20; US, ultrasound, range 0–18.

**Figure 2:**
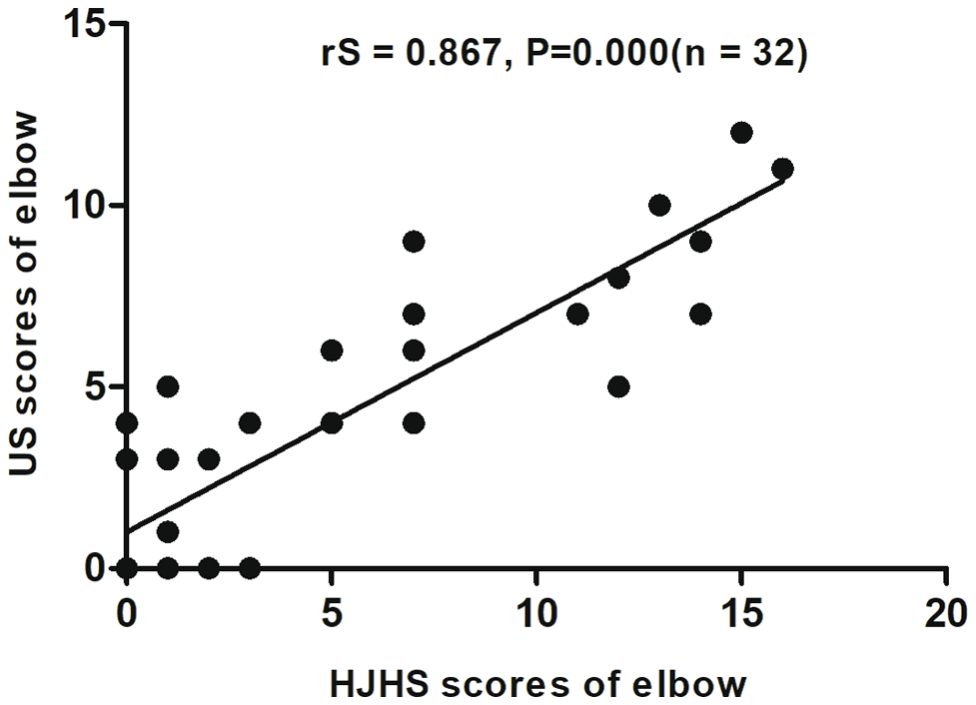
Scatter plot showing the correlation between the clinical Hemophilia Joint Health Score (HJHS 2.1) and ultrasound (US) score for the elbow.

**Figure 3:**
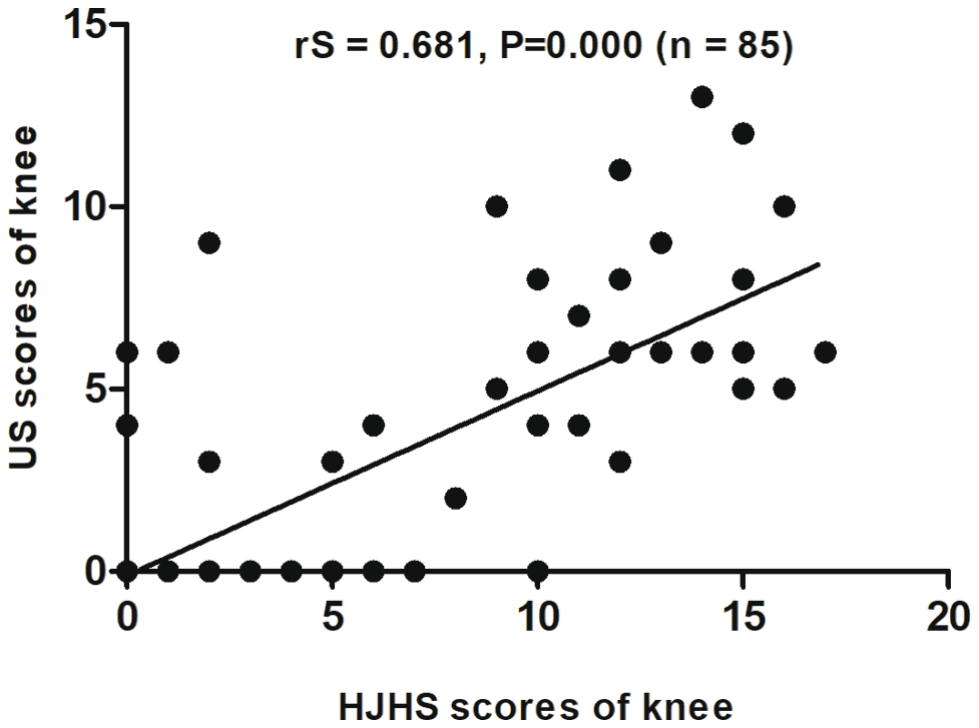
Scatter plot showing the correlation between the clinical Hemophilia Joint Health Score (HJHS 2.1) and ultrasound (US) score for the knee.

**Figure 4:**
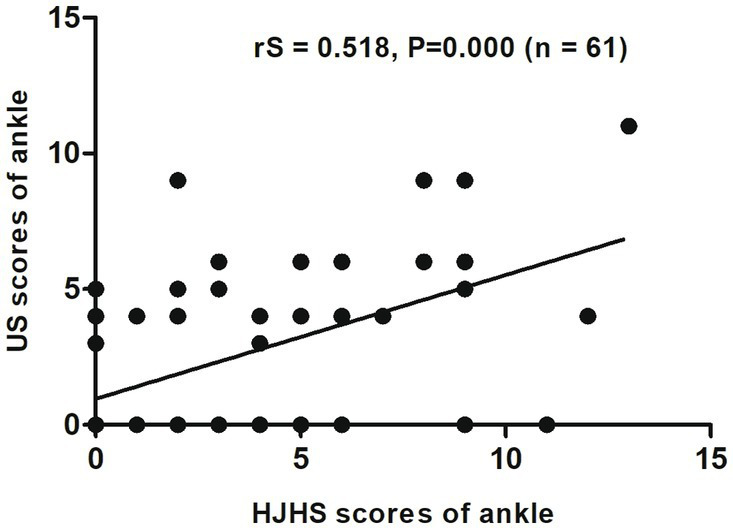
Scatter plot showing the correlation between the clinical Hemophilia Joint Health Score (HJHS 2.1) and ultrasound (US) score for the ankle.

When different joints were evaluated separately, an excellent correlation was found between HJHS 2.1 and US scores for the elbows (rS = 0.867, *p* = 0.000, CI: 0.709–0.941) (Figure 2) and a substantial correlation was found for the knees (rS = 0.681, *p* = 0.000, CI: 0.527–0.797) (Figure 3). On the other hand, the correlation between the scores for the ankles was relatively moderate (rS = 0.518, *p* = 0.000, CI: 0.308–0.705) (Figure 4).

Five subjects (20.8%, 5/24 joints) had normal HJHS 2.1 for ankles but showed signs of haemophilic arthropathy in US assessments (3 points for two subjects, 4 points for two subjects, and 5 points for one subject). All of these five joints had a synovial thickness greater than 2.5 mm (scoring 3 points), of which two showed synovial vascular hyperplasia and one had both synovial vascular hyperplasia and hemosiderin deposition (Figure 4).

In contrast, 50 subjects (44.1%, 15/34 joints) had normal US ankle scores but showed abnormal findings in HJHS 2.1 assessments (1 point for four subjects, 2 points for three subjects, 3 points for one subject, 4 points for three subjects, 5 points for one subject, 6 points for one subject, 9 points for one subject, and 11 points for one subject) (Figure 4). Six joints showed mild muscle atrophy while three had severe change. Among these, two joints showed crepitus during activity and decreased curvature, while one showed a decrease in extension and a change in joint strength based on the above changes. Mild and severe swelling was found in four joints each (three of these eight joints showed swelling in combination with muscle atrophy), of which seven had shown swelling for ≥6 months; one subject had a simple joint strength change that was not detected by US. Among the patients who reported a swelling duration of ≥6 months, one had joint pain and another had both joint pain and joint strength changes. Moreover, among the three patients with both muscle atrophy and swelling, one had joint pain, one experienced reduced stretching, and the third had a decreased curvature, decreased extension, and joint strength.

## 4. Discussion

Repeated joint bleeding in haemophilia often leads to joint deformity and dysfunction, which seriously affects the quality of life of patients (9), and with age, these lesions become more apparent (10). US has recently attracted growing interest as a potential tool to assess joint status and identify early arthropathic changes in haemophilia patients, which can ensure initiation of treatment as soon as possible and thereby prevent joint damage (11–13).

Previous studies showed a strong correlation between HJHS and US in the evaluation of the joints, and US could reveal a higher percentage of abnormalities than HJHS in both children and adults (7, 11, 14, 15). However, Poonnoose et al. (5) suggested that the correlation between HJHS and US for the osteochondral component was moderate (rS = 0.45) while that for the soft tissue component was poor (rS = 0.26).

In our study, we found a good correlation between HJHS 2.1 and US for all study joints (rS = 0.651, *p* = 0.000, CI: 0.553–0.763). This was consistent with the findings obtained by Altisent et al. (16). Early identification of synovial tissue thickening is important to prevent progression of haemophilic osteoarthrosis since the thickened synovial membrane and fragile neovascularization can increase the chance of joint bleeding and directly destroy articular cartilage, which further leads to the occurrence and progression of haemophilic arthropathy. In this study, the US scores for 15.5% of the joints that did not show abnormalities on HJHS 2.1 indicated haemophilic arthropathy, and synovial thickening was identified in all of these patients. The thickening was partial with small changes in the cartilage and hemosiderin deposition, which suggests that the US scoring system could identify early pathological changes in joints appearing normal in clinical examination and offers advantages over HJHS 2.1 in assessments of synovial thickening, hemosiderin deposition and cartilage changes. Due to the convenience of US, in the case of routine monitoring of joint function or even acute joint bleeding in hemophilia patients, US can provide clinical diagnosis and treatment with more rapid, convenient, and accurate therapeutic guidance value. Especially in the process of dynamic monitoring of joint disease changes in hemophiliacs, more sensitive intervention treatment can be given to hemophiliacs according to the results of dynamic changes in US, which can prevent further deterioration of the joint disease. However, our study also revealed changes with HJHS 2.1 that were not detected by the US scoring system and may require physical or drug intervention, which showed a higher percentage (50.5%) of positive points on HJHS 2.1 in relation to muscle atrophy, joint strength changes, or swelling while US scores for these findings were negative in previous studies (6, 16). This poor specificity of US in soft-tissue findings suggests that US is still lacking in the comprehensive evaluation of joint soft tissue changes and that the US scoring system and HJHS 2.1 would complement each other in order to ensure better assessment of joint status.

For a single joint US evaluation, Aspdahl et al. (14) suggested that a strong correlation exists between the HJHS and the US scores for elbows and knees (rS = 0.57, *p* < 0.01 and rS = 0.76, *p* < 0.01), but the correlation for ankles was substantially weaker (rS = 0.36, *p* = 0.04). In comparison with their results, our study showed a significant correlation between HJHS and US scores in all of the elbow, knee and ankle joints (rS = 0.867, *p* = 0.000; rS = 0.681, *p* = 0.000 and rS = 0.518, *p* = 0.000, respectively). This may be attributable to the size of our research sample. However, in our study, the correlation for the ankle joints was more moderate than that for the elbow and ankle joints, which was the same trend as that observed in their study. This suggests that when evaluating the osteoarticular lesions of the ankle joint, there is a strong need for a comprehensive evaluation that includes both US and HJHS assessments.

## 5. Conclusion

Our study confirmed that in paediatric patients with haemophilia A, the US scoring system correlated well with HJHS 2.1 assessments for overall and individual joint assessments, with the correlations being excellent for elbows, substantial for knees, and moderate for ankles. US could identify early pathological changes in apparently healthy joints and showed advantages over the HJHS 2.1 in terms of synovial thickening, hemosiderin deposition, and cartilage changes. However, it still had defects that prevented it from replacing the HJHS. Instead, it can serve as an imaging examination complementing HJHS 2.1 assessments.

## Data availability statement

The original contributions presented in the study are included in the article/supplementary material, further inquiries can be directed to the corresponding authors.

## Ethics statement

The studies involving human participants were reviewed and approved by Affiliated Hospital of Guizhou Medical University. Written informed consent to participate in this study was provided by the participants’ legal guardian/next of kin.

## Author contributions

YLi, FW, YLiu, and DT conceived of and designed the study. They had full access to all data in the study, and take responsibility for the integrity of the data, the accuracy of the data analysis, and the writing of the report. XZ, JW, and ZH critically revised the report. CP, JZ, QZ, LB, and LS performed the statistical analyses. YLi, FW, CP, JZ, QZ, LB, LS, JW, ZH, XZ, DT, and YLiu contributed to the data acquisition and analyses. All authors contributed to the article and approved the submitted version.

## Funding

This study was supported by the National Natural Science Foundation of China (nos. 82160519 and 31660326); the Natural Science Foundation of Guizhou Province [nos. QianKeHe-ZK (2023) Key 042 and QianKeHe Support (2022)181]; and the Natural Science Foundation of Guiyang City [nos. (2022)4-3-2 and (2022)4-3-10]. The funders of the study had no role in study design, data collection, data analysis, data interpretation, or writing of the report.

## Conflict of interest

The authors declare that the research was conducted in the absence of any commercial or financial relationships that could be construed as a potential conflict of interest.

## Publisher’s note

All claims expressed in this article are solely those of the authors and do not necessarily represent those of their affiliated organizations, or those of the publisher, the editors and the reviewers. Any product that may be evaluated in this article, or claim that may be made by its manufacturer, is not guaranteed or endorsed by the publisher.
